# Meteorological Report

**Published:** 1879-09

**Authors:** 


### METEOROLOGICAL REPORT FOR AUGUST, 1879.

| RAIN. DATE | RAIN. IN. | Mean daily Barometer. | Mean daily Thermometer | Mean daily Humidity. | Maximum Thermometer | Minimum Thermometer | Prevailing direction. |
|---|---|---|---|---|---|---|---|
| 1 | 80 | 20.138 | 71.0 | 90.0 | 78 | 69 | S. E. |
| 2 | 56 | 30.124 | 72.0 | 90.0 | 78 | 68 | E. |
| 3 | 02 | 30.043 | 75.7 | 78.0 | 84 | 71 | E. |
| 4 | 0 | 29.955 | 80.7 | 68.0 | 88 | 70 | W. |
| 5 | 0 | 29.947 | 82.5 | 69.3 | 89 | 74 | S. W. |
| 6 | 32 | 29.933 | 80.5 | 77.7 | 91 | 75 | S. W. |
| 7 | 0 | 29.871 | 80.7 | 74.0 | 88 | 75 | W. |
| 8 | 43 | 29.856 | 77.0 | 75.7 | 85 | 72 | W. |
| 9 | 0 | 29.912 | 69 5 | 66.0 | 77 | 66 | N. W. |
| 10 | 0 | 30 032 | 69.5 | 63.3 | 75 | 62 | E. |
| 11 | 0 | 30.065 | 71.5 | 62.0 | 79 | 62 | E. |
| 12 | 0 | 30.092 | 73.2 | 56.7 | 78 | 63 | E. |
| 13 | 0 | 30.032 | 73.7 | 66.0 | 81 | 64 | E. |
| 14 | 03 | 29.954 | 73.2 | 79 0 | 78 | 70 | W. |
| 15 | 01 | 29.843 | 75.5 | 78.7 | 81 | 70 | S. E. |
| 16 | 55 | 29.807 | 76.0 | 78.7 | 85 | 70 | S. E. |
| 17 | 06 | 29.823 | 71.7 | 82.0 | 78 | 68 | W. |
| 18 | 0 | 29.878 | 71 0 | 78.0 | 79 | 62 | W. |
| 19 | 0 | 29.975 | 75.0 | 67.0 | 83 | 63 | E. |
| 20 | 01 | 29.998 | 75.0 | 78.7 | 84 | 68 | E. |
| 21 | 0 | 30.005 | 75.7 | 75.0 | 83 | 66 | E. |
| 22 | 01 | 29.924 | 74.0 | 83.3 | 81 | 70 | E. |
| 23 | 42 | 29.791 | 75.0 | 86.3 | 81 | 71 | S. E. |
| 24 | 1.13 | 29.723 | 77.7 | 82.3 | 83 | 73 | S. |
| 25 | 41 | 29.838 | 74.2 | 70.3 | 80 | 70 | S. W. |
| 26 | 0 | 29.897 | 65.2 | 66.3 | 74 | 59 | N. W. |
| 27 | 0 | 29.927 | 67.5 | 64.7 | 75 | 57 | N. E. |
| 28 | 0 | 30.017 | 67.5 | 65.3 | 75 | 60 | M. E. |
| 29 | 0 | 30.087 | 69.0 | 64.0 | 77 | 58 | N. E. |
| 30 | 0 | 30.031 | 70.0 | 66.3 | 78 | 62 | N. E. |
| 31 | 0 | 29 914 | 74.5 | 68 3 | 83 | 63 | N. E. |
| Monthly Means. | | 29.949 | 73.7 | 73.2 | 80.9 | 66.8 | |

Highest Barometer, 30.165 on the 1st.
Lowest Barometer, 29.674, on the 24th.
Monthly range of Barometer, 0.491.
Highest Temp. 91°, on 6th, Lowest Temp. 57°, on 27th.
Monthly range of Temperature, 34°.
Greatest daily range of Temperature, 20°, on the 19th and 31st.
Least daily range of Temperature, 8°, on the 14th.
Mean of Maximum Temperatures, 80.9.
Mean of Minimum Temperatures, 66.8.
Mean daily range of Temperature, 14.1. Total rainfall, 4.76 inches.
Prevailing wind, East. Total movement of wind, 61.46 miles.
Maximum velocity of wind and direction, 27 miles per hour, South, 23d.
Number Cloudy days on which rain fell, 10.
Number of cloudy days on which no rain fell, 7.
Total number of days on which rain fell, 18.

H. HALL, Corp'l. Signal Service U.S. A.

Atlanta, Ga., Sept. 1, 1879.

